# Comprehensive identification of proteins interacting with long non-coding RNA TUG1 in R-loop regulation

**DOI:** 10.1093/jb/mvaf042

**Published:** 2025-07-14

**Authors:** Jingqi Xie, Miho M Suzuki, Kenta Iijima, Keiko Shinjo, Tatsunori Nishimura, Shinya Watanabe, Reiko Nakagawa, Tatsuo Ito, Yutaka Kondo

**Affiliations:** Division of Cancer Biology, Nagoya University Graduate School of Medicine, 65 Tsurumai-cho, Showa-ku, Nagoya, Aichi 466-8550, Japan; Division of Cancer Biology, Nagoya University Graduate School of Medicine, 65 Tsurumai-cho, Showa-ku, Nagoya, Aichi 466-8550, Japan; Laboratory Animal Facilities and Services, Institute of Photonics Medicine, Hamamatsu University School of Medicine, 1-20-1 Handayama, Higashi-ku, Hamamatsu, Shizuoka 431-3192, Japan; Division of Cancer Biology, Nagoya University Graduate School of Medicine, 65 Tsurumai-cho, Showa-ku, Nagoya, Aichi 466-8550, Japan; Division of Cancer Biology, Nagoya University Graduate School of Medicine, 65 Tsurumai-cho, Showa-ku, Nagoya, Aichi 466-8550, Japan; Division of Cancer Biology, Nagoya University Graduate School of Medicine, 65 Tsurumai-cho, Showa-ku, Nagoya, Aichi 466-8550, Japan; Laboratory for Cell-Free Protein Synthesis, RIKEN Center for Biosystems Dynamics Research, 6-7-1 Minatojima-minamimachi, Chuo-ku, Kobe, Hyogo 650-0047, Japan; Department of Hygiene, Kawasaki Medical University, 577 Matsushima, Kurashiki, Okayama 701-0192, Japan; Division of Cancer Biology, Nagoya University Graduate School of Medicine, 65 Tsurumai-cho, Showa-ku, Nagoya, Aichi 466-8550, Japan; Institute for Glyco-core Research (iGCORE), Nagoya University, Furo-cho, Chikusa-ku, Nagoya, Aichi 464-8601, Japan; Center for One Medicine Innovative Translational Research (COMIT), Nagoya University, Furo-cho, Chikusa-ku, Nagoya, Aichi 464-8601, Japan

**Keywords:** long non-coding RNA, proteomics, R-loop, RNA-binding proteins, TUG1

## Abstract

Long non-coding RNAs (lncRNAs) regulate a wide array of cellular processes through interactions with RNA-binding proteins (RBPs). Taurine Upregulated Gene 1 (TUG1) is an lncRNA that is overexpressed in many types of cancer and has been implicated in resolving R-loops, thereby maintaining genomic integrity. However, the full spectrum of its protein interactions and stress-responsive dynamics remains unclear. Here, we employed CRISPR-assisted RNA-protein interaction detection (CARPID) combined with mass spectrometry to comprehensively identify the interacting proteins of TUG1 in HEK293T cells. Using three distinct single-guide RNAs (sgRNAs) targeting different regions of TUG1, we consistently identified 17 TUG1-interacting proteins under basal conditions. Upon camptothecin (CPT) treatment, which induces R-loop formation, the number of associated proteins increased to 25. Under these stress conditions, the protein sets identified by each sgRNA showed greater overlap, suggesting a more conserved pattern of TUG1-protein interactions in response to R-loop accumulation. Many of these proteins are known R-loop-associated factors, including DEAD/DEAH-box RNA helicases, poly(ADP-ribose) polymerase 1 (PARP1) and heterogeneous nuclear ribonucleoproteins (HNRNPs), indicating that TUG1 engages R-loop regulatory machinery to maintain genome integrity. Our study provides new insights into lncRNA-mediated R-loop regulation and its role in genome maintenance.

## Graphical Abstract

**Graphical Abstract:**
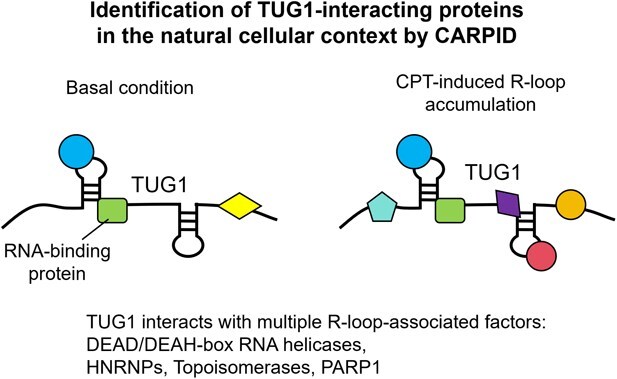

## Abbreviations

ASO: Antisense oligonucleotide
CARPID: CRISPR-assisted RNA-protein interaction detection
ChIRP: Chromatin isolation by RNA purification
CPT: Camptothecin
DHX9: DEAH-box helicase 9
FISH: Fluorescent *in situ* hybridization
GO: Gene ontology
HNRNP: Heterogeneous nuclear ribonucleoprotein
lncRNA: Long non-coding RNA
MALAT1: Metastasis-associated lung adenocarcinoma transcript 1
MDC1: DNA damage checkpoint 1
MS: Mass spectrometry
PARP1: Poly (ADP-ribose) polymerase 1
RBP: RNA-binding protein
RPA: Replication protein A
sgRNA: Single-guide RNA
SMARCA5: SWI/SNF-related matrix-associated actin-dependent regulator of chromatin subfamily A member 5
TUG1: Taurine upregulated 1

Non-coding RNAs are a broad class of transcripts that lack protein-coding capacity and are broadly expressed in human cells (*1**,*  *2*). Among them, long non-coding RNAs (lncRNAs), defined as transcripts longer than 200 nucleotides, have emerged as key regulators of diverse biological processes (*3**,*  *4*). In particular, nuclear-localized lncRNAs influence chromatin structure, modulate epigenetic marks, regulate gene expression and contribute to DNA repair (*3**,*  *5*–*8*).

The functional versatility of lncRNAs primarily exerted through interactions with RBPs, forming dynamic ribonucleoprotein complexes that facilitate their biological roles (*9*–*13*). By interacting with RBPs, lncRNAs not only regulate their own stability and function but also control the recruitment, localization and activity of RBPs (*14*–*16*). These interactions fine-tune gene expression and play a crucial role in maintaining cellular homeostasis (*15*). Given their essential functions, comprehensively identifying lncRNA-interacting proteins is vital for understanding the molecular mechanisms underlying lncRNA function.

Taurine Upregulated Gene 1 (TUG1) is an lncRNA that has been extensively implicated in cancer progression and chemoresistance (*17*–*19*). We have recently shown that TUG1 is rapidly upregulated under replication stress via the ATR-CHK1 signalling pathway, and subsequently interacts with key RBPs such as replication protein A (RPA) and DEAH-box helicase 9 (DHX9) (*20*). Through these interactions, TUG1 facilitates the resolution of R-loops—three-stranded nucleic acid structures composed of an RNA/DNA hybrid and displaced single-stranded DNA. The accumulation of R-loops has been associated with replication stress and subsequent DNA damage (*21*–*23*). Notably, depletion of TUG1 in cancer cells leads to excessive R-loop accumulation, prolonged replication stress and increased apoptosis, ultimately inhibiting cell proliferation (*20*).

Despite the emerging importance of TUG1 in R-loop regulation, the full repertoire of TUG1-interacting proteins remains largely unknown. Conventional RNA-protein interaction mapping techniques, such as chromatin isolation by RNA purification (ChIRP) or affinity purification-mass spectrometry (MS), have provided valuable insights into lncRNA-protein interactions but are often limited by crosslinking biases, indirect interactions, and the inability to capture dynamic or weakly interacting proteins (*24*). To overcome these challenges, we employed CRISPR-assisted RNA-protein interaction detection (CARPID), a technique that enables the specific labelling and identification of proteins interacting with target lncRNAs in living cells (*25*). CARPID utilizes a catalytically inactive Cas13 (dCasRx) (*26*) fused with an engineered biotin ligase (BASU) (*27*) to selectively label proteins in close proximity to the target RNA without requiring chemical crosslinking. This method allows for the high-specificity capture of RBPs while minimizing background noise (*25*).

In this study, we used CARPID to systematically identify TUG1-interacting proteins in HEK293T cells, under both normal conditions and in response to camptothecin (CPT)-induced replication stress, which promotes R-loop accumulation. Using three distinct single-guide RNAs (sgRNAs), we identified a set of TUG1-associated proteins in both conditions. A substantial proportion of these proteins are known R-loop regulators. These findings suggest that TUG1 dynamically modulates its protein interactions in response to replication stress, reinforcing its role in R-loop resolution and genomic maintenance. By integrating CARPID with MS, our study provides a comprehensive proteomic landscape of TUG1-associated proteins, offering new insights into the molecular mechanisms of lncRNA-mediated R-loop regulation.

## Materials and Methods

### Cell culture and transfection

HEK293T cells (RIKEN Cell Bank, Japan) were cultured in Dulbecco’s modified Eagle’s medium supplemented with 10% foetal bovine serum and 1x Antibiotic-Antimycotic (Gibco, Thermo Fisher Scientific, MA, USA), at 37°C with 5% CO_2_. Transfections of plasmids and antisense oligonucleotides (ASOs) were conducted using Lipofectamine 3000 (Thermo Fisher Scientific) or ScreenFect A Plus (FUJIFILM Wako Pure Chemical, Japan) following the manufacturer’s instructions. Plasmids and ASOs used are shown in Supplementary Table 1.

### Cell proliferation assay

A total of 2 × 10^5^ cells were seeded per well in six-well plates. After 24 h, cells were transfected with ASO. Cells were harvested by trypsinization at different time points, and cell numbers were determined by manual counting.

### Single-molecule fluorescent *in situ* hybridization

Cells were seeded in 96-well half-area film bottom microplates (Corning, NY, USA, Cat. #4680) and incubated for 24 h. Single-molecule fluorescent in situ hybridization (FISH) experiment was conducted using the ViewRNA Cell Plus Assay Kit (Thermo Fisher Scientific) following the manufacturer’s instructions. TUG1 transcripts were detected using a type1 probe set mixture of exon 1 (ViewRNA Probe Set; Assay ID: VA1-6000856), exon 2 (ViewRNA Probe Set; Assay ID: VA1-11879), and exon 3 (ViewRNA Probe Set; Assay ID: VPMFW2G), in a 1:1:1 ratio. Cells were counterstained with 4′,6-diamidino-2-phenylindole (DAPI, Cell Signaling Technology, MA, USA). Images obtained with a confocal microscope TiE-A1R (Nikon, Japan) were analysed by NIS-Elements viewer software (Nikon).

### R-loop detection via slot blot

Total nucleic acid was extracted from cell nuclei using NucleoSpin Tissue kits (MACHEREY-NAGEL, Germany). The purified DNA was treated with or without 1 U of RNase H (New England Biolabs) and incubated overnight at 37°C. The samples were subsequently applied to positively charged Nylon Membranes (Roche, Switzerland), assembled within the BioDot-SF microfiltration apparatus (Bio-Rad, CA, USA), using TBS buffer (10 mM Tris pH 7.5, 150 mM NaCl). The blotted DNA was crosslinked to the membrane using a Stratalinker UV Crosslinker 2400 (Stratagene, CA, USA) at 120 mJ/cm^2^. RNA/DNA hybrids were detected with the Anti-RNA/DNA hybrid antibody, clone S9.6 (MABE1095, Merck Millipore, 1:1000 dilution). The membrane was subsequently stained with 1x SYBR™ Gold Nucleic Acid Gel Stain (Thermo Fisher Scientific) in TBS-T (TBS buffer with 0.1% Tween 20) to normalize the DNA quantity. After sequential washes with TBS-T and TBS, performed three times each, fluorescent images were captured using the ChemiDoc Touch MP imaging system (Bio-Rad).

### Design of sgRNA

The sgRNAs were designed as previously described by Bandaru *et al*. (*28*). Structural information for TUG1 was obtained from the NONCODE database (NONCODE ID: NONHSAT084833.2) (*29*), based on inferences from dimethyl sulfate sequencing (DMS-seq) probing (Dataset source: Control_Fibroblast-Vivo_Fibrob). The design of TUG1 sgRNAs was performed using SnapGene software (version 4.1.9) (https://www.snapgene.com/). To ensure target specificity, a similarity search against the entire human genome was conducted using GGGenome Blast (https://gggenome.dbcls.jp/). sgRNAs exhibiting self-complementarity or dimerization potential were excluded. The designed sgRNAs targeting single-stranded and double-stranded regions of TUG1 are listed in Supplementary Table 1. For negative control, a non-targeting sgRNA (sgNoTarget) (*26*), which does not target any known human genes, was employed. Additionally, the sgRNA sequence for Metastasis-associated lung adenocarcinoma transcript 1 (MALAT1) (sgMALAT1) was adapted from Yi *et al*. (*25*).

### sgRNA-expressing vector construction

Oligonucleotides with BbsI restriction enzyme recognition sequences added to the 5′ and 3′ ends of the designed sgRNA sequence were synthesized. These sgRNA oligos were inserted into the CasRx pre-gRNA cloning backbone vector (a gift from Patrick Hsu, Addgene plasmid # 109054) (*26*) at the BbsI sites.

### Assessment of sgRNA cleavage efficiency

The sgRNA cleavage efficiency was examined using a previously established protocol (*26*). Cells were plated at a density of 1 x 10^5^ cells per well in a 24-well plate. After 24 h, the transfection was performed with 250 ng of the EF1a-CasRx-2A-EGFP plasmid and 250 ng of a sgRNA-expressing vector per well, using Lipofectamine 3000. Cells were harvested 48 h post-transfection, and sgRNA-mediated cleavage efficiency was assessed via reverse transcription quantitative polymerase chain reaction (RT-qPCR). The assessment involved comparing the amplification results of the sgRNA-treated groups with those of the sgNoTarget group. Oligonucleotide primers used for RT-qPCR are detailed in Supplementary Table 1.

### CARPID

The CARPID was performed following a previously established protocol (*25*) with some modifications. A total of 4 × 10^6^ cells were seeded in a 100-mm collagen-coated dish (4020–010, IWAKI, Japan) and cultured for 24 h before the assay. Cells were transfected with 4 μg of CARPID BASU-dCasRx plasmid and 4 μg of sgRNA-expressing vector per dish using ScreenFect A Plus. Forty-eight hours post-transfection, cells were incubated with 200 μM biotin (Tokyo Chemical Industry, Japan) for 15 min and washed three times with cold PBS. The cell pellets were resuspended in 1× PBS/1 mM EDTA solution to remove extraneous contaminants. Subsequently, the plasma membranes were lysed by resuspending the pellets in ice-cold TritonX-100 lysis buffer (10 mM Tris–HCl pH 7.5, 0.03% TritonX-100, 150 mM NaCl) for 5 min. After centrifugation at 4°C and 14,000 rpm for 10 min, the resulting pellet, representing the nuclear fraction, was collected. The nuclear fraction was further lysed in 1 ml lysis buffer (50 mM Tris–HCl, pH 7.4, 150 mM NaCl, 0.5% Triton X-100, 1 mM EDTA supplemented with protease inhibitors). Following rotation at 4°C for 20 min, the supernatants were collected via centrifugation, and the protein concentrations were equalized. Biotinylated proteins were isolated using Dynabeads MyOne Streptavidin T1 (Thermo Fisher Scientific) and eluted into elution buffer (125 mM Tris–HCl, pH 7.4, 4% SDS, 20% glycerol, 0.04% Bromophenol Blue, 8% 2-Mercaptoethanol, 5 mM Biotin) by incubation for 10 min at 95°C. The protein samples were separated on SDS-PAGE gels and detected using Silver Stain KANTO III (KANTO CHEMICAL, Japan), western blotting or MS analysis, which is described in the following section.

### Nano-liquid chromatography–tandem mass spectrometry

The proteins in each gel slice underwent a series of treatments: they were first reduced using 10 mM dithiothreitol (DTT) at 56°C for 1 h, followed by alkylation with 55 mM iodoacetamide at room temperature for 45 min in the absence of light. Subsequently, digestion was performed using 10 μg/ml modified trypsin (Pierce, MS Grade, Thermo Fisher Scientific) at 37°C for 16 h. The resulting peptides were then extracted using a solution of 1% trifluoroacetic acid and 50% acetonitrile, followed by vacuum drying and dissolution in a solution containing 2% acetonitrile and 0.1% formic acid. The mass spectra were acquired using an LTQ-Orbitrap Velos Pro (Thermo Fisher Scientific) coupled to a nanoflow UHPLC system (ADVANCE UHPLC, AMR Inc., Japan) equipped with an Advanced Captive Spray SOURCE (AMR Inc.). The peptide mixtures were initially loaded onto a C18 ID 0.1 × 20 mm, 5 μm (particle size) trap column (Acclaim PepMap 100 C18, Thermo Fisher Scientific) and subsequently fractionated by a C18, ID 0.075 × 500 mm, particle size 3 μm column (CERI, Japan). Peptides were eluted at a flow rate of 300 nl/min using a linear gradient of 5–35% solvent B (100% acetonitrile) in solvent A (100% H_2_O, 0.1% formic acid) over 60 min. The mass spectrometer was programmed to perform 11 successive scans. The first scan consisted of a full-scan MS over the range of 350–1800 m/z using the orbitrap at a resolution of 60,000. The subsequent scans (second to 11th) were carried out by the ion trap analyser with automatic data-dependent tandem mass spectrometry (MS/MS) scans of the top 12 most abundant ions identified in the first scan. MS/MS spectra were generated using a normalized collision energy of 35% CID, with a 2-m/z isolation width and an exclusion time of 90 s for molecules within the same m/z value range.

### Mass spectrometry data analysis

The raw data files were searched against the *Homo sapiens* dataset (Uniprot Proteome *H. sapiens* UP000005640, 2022_05_25 downloaded) with the common Repository of Adventitious Proteins (cRAP, ftp://ftp.thegpm.org/fasta/cRAP) using MASCOT version 2.6 (Matrix Science, Japan) via Proteome Discoverer 2.5.0.400, with a false discovery rate (FDR) set at 0.01. For the search parameters, precursor mass tolerance and product tolerance were set at 10 ppm and 0.8 Da, respectively. Carbamidomethylation of cysteine was specified as a static modification, while oxidation of methionine and acetylation of protein N-terminal were defined as variable modifications. The allowed number of missed cleavage sites was limited to two. Label-free quantification was performed using Proteome Discoverer, and enrichment analysis was applied to proteins identified with two or more peptides. Abundance values from label-free quantification were used to validate MS/MS-based peptide and protein identifications. Any missing values were imputed with values representing the detection limit of the mass spectrometer. CARPID experiments were performed in three independent biological replicates for each of the three sgRNAs targeting TUG1, sgRNA targeting MALAT1 and the sgNoTarget.

The rank products test (*30*) was employed to identify proteins that exhibited statistically significant enrichment in the samples expressing TUG1 sgRNA, compared to those expressing NoTarget sgRNA controls. Proteins were deemed significantly enriched if they had an adjusted *P*-value ≤0.05 and a peptide abundance difference >1e+07. This computational method was also applied to analyse proteins associated with lncRNA MALAT1.

### Gene ontology analysis

Gene ontology (GO) analysis was performed using Enrichr (https://maayanlab.cloud/Enrichr/) (*31*). The top five significantly overrepresented biological processes were identified based on Fisher’s exact test and ranked according to their Benjamini–Hochberg corrected *P*-values.

### Statistical analysis

The results are presented as the mean ± standard error of the mean (SEM) or standard deviation (SD), as specified. The number of times experiments were replicated with similar outcomes and the sample size (*n*) for statistical evaluations are detailed in the figure legends. Statistical analysis was conducted using Microsoft Excel, GraphPad Prism 10 and R. *P*-values are indicated as follows: ns (not significant), *P* > 0.05, ^*^P < 0.05, ^**^P < 0.01 and ^***^P < 0.001.

**Fig. 1:**
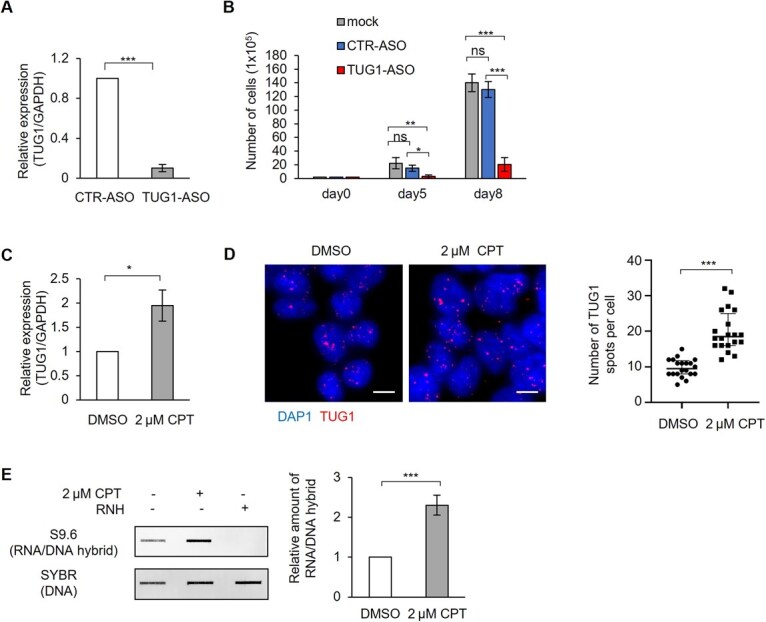
**Expression of TUG1 is induced under replication stress.** (A) RT-qPCR analysis of TUG1 knockdown in HEK293T cells 4 h after ASO transfection. Mean ± SD, *n* = 3. (B) Cell proliferation at 5 and 8 days post-seeding following CTR-ASO or TUG1-ASO transfection. Mean ± SD, *n* = 3. ns, *P* ≥ 0.05; ^*^P < 0.05; ^**^P < 0.01; ^***^P < 0.001, two-sided *t*-test. (C) TUG1 expression after DMSO or 2 μM CPT treatment (30 min), normalized to GAPDH (RT-qPCR, *n* = 3). ^*^P < 0.05, two-sided *t*-test. (D) Left, representative TUG1 RNA FISH images after DMSO or CPT treatment. Nuclei were stained with DAPI. Scale bar = 10 μm. Right, quantification of RNA FISH experiments. The y-axis indicates the number of TUG1 spots per cell. ^***^P < 0.001, two-sided *t*-test. (E) Left, RNA/DNA hybrid slot blot using S9.6 antibody. Cells were treated with 2 μM CPT for 30 min. Genomic DNA was pre-treated with RNase H. SYBR Gold: loading control. Right, quantification of S9.6 signal. Mean ± SD, *n* = 3. ^***^P < 0.001, two-sided *t*-test.

**Fig. 2:**
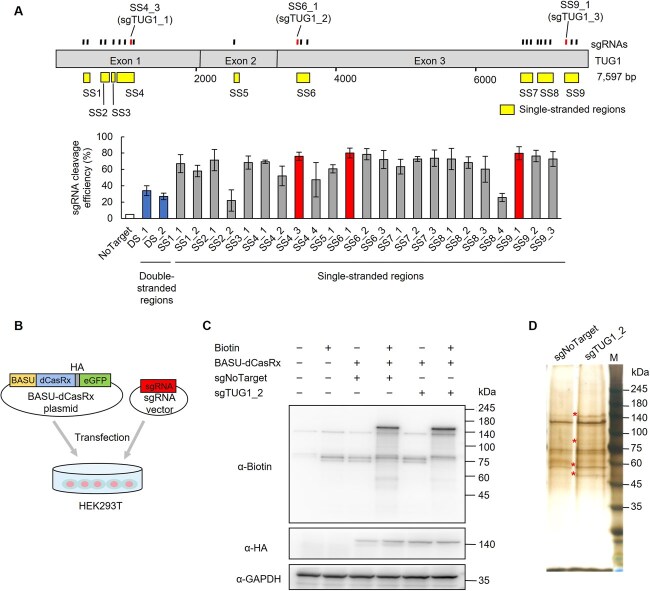
**Design, selection and validation of sgRNAs for CARPID-based protein identification.** Top, schematic of the TUG1 transcript with predicted single-stranded (SS) regions (yellow squares). Twenty-five sgRNAs targeting nine SS regions are shown above. The three most efficient sgRNAs—sgTUG1_1 (SS4_3), sgTUG1_2 (SS6_1) and sgTUG1_3 (SS9_1)—are indicated by vertical lines above the corresponding regions.. Bottom, RT-qPCR showing sgRNA-mediated cleavage efficiency in double-stranded (DS) and SS regions, normalized to GAPDH. sgNoTarget: negative control. Mean ± SD, *n* = 3. (B) CARPID workflow schematic. BASU, biotin ligase; dCasRx, catalytically inactive Cas13; HA, HA tag; eGFP, enhanced GFP. (C) Western blot of biotin-treated and untreated cells. Biotinylated proteins were detected using a biotin antibody; BASU-dCasRx expression was confirmed using an HA antibody. GAPDH: loading control. (D) Silver-stained gel of CARPID pull-down samples using sgNoTarget or sgTUG1_2. M, molecular weight marker (kDa). Specific bands enriched in sgTUG1_2 sample are marked with asterisks (*).

## Results

### TUG1 expression is induced under replication stress in HEK293T cells

To establish the CARPID workflow, we chose HEK293T cells, which were originally used to develop CARPID (*25*), because their high transient-transfection efficiency is essential for achieving robust expression of the multiple plasmids required for successful proximity labelling. Although the role of TUG1 in cancer cells has been elucidated (*20*), in this study we focused on analysing the proteins that associate with TUG1 under replication stress in normal cells. TUG1-targeting ASO reduced TUG1 transcript levels to <10% within 4 h, significantly impairing cell proliferation at both 5 and 8 days compared to control ASO (CTR-ASO) (Fig. 1A and B). These findings are consistent with previous reports in cancer cells, where TUG1 depletion also resulted in reduced proliferation (*20*). Notably, CPT-induced replication stress led to a 2-fold increase in TUG1 transcript levels compared to DMSO-treated controls (Fig. 1C). Single-molecule RNA FISH further confirmed this upregulation, showing increased TUG1 spot numbers upon CPT treatment (Fig. 1D). HEK293T cells, although derived from human kidney cells, contain SV40 T antigen, which promotes rapid proliferation (*32**,*  *33*). Given their fast growth, they provide a suitable model for investigating the role of TUG1 in replication stress responses. To assess the extent of R-loop formation in HEK293T cells following CPT treatment, we performed an S9.6 antibody-based slot blot assay to quantify RNA/DNA hybrids (*34*). While basal R-loop levels were detectable in untreated cells, CPT treatment led to a 2.3-fold increase in S9.6 signal intensity. RNase H treatment, which specifically degrades RNA/DNA hybrids, abolished the S9.6 signal, confirming the specificity of the antibody for RNA/DNA hybrids (Fig. 1E).

### Selection of sgRNAs and validation of CARPID for TUG1 interactome profiling

To select sgRNAs for CARPID targeting TUG1, we first tested cleavage efficiency. A total of 25 sgRNAs were designed across nine single-stranded regions of TUG1, as Cas13 nuclease activity depends on RNA secondary structure and preferentially cleaves single-stranded regions (Fig. 2A) (*28*). HEK293T cells were co-transfected with plasmids expressing each sgRNA along with nuclease-active CasRx. Among all candidates, SS4_3, SS6_1 and SS9_1 exhibited the highest cleavage efficiency (>80%) (Fig. 2A). These sgRNAs were designated as sgTUG1_1, sgTUG1_2 and sgTUG1_3, respectively, and were used for subsequent CARPID experiments. For CARPID, HEK293T cells were co-transfected with sgRNA-expressing vectors and BASU-dCasRx plasmids, which enable targeted biotinylation using catalytically inactive Cas13 (dCasRx) fused to an engineered BASU biotin ligase (BASU-dCasRx) (Fig. 2B). After a 15-min biotin treatment, biotinylated nuclear proteins were isolated via streptavidin pulldown. Western blot analysis confirmed efficient biotinylation, while HA antibody verified BASU-dCasRx expression (Fig. 2C). Silver staining of streptavidin-captured proteins revealed specific bands in sgTUG1 samples that were absent in sgNoTarget controls (Fig. 2D). These differentially enriched proteins were subsequently identified by MS (Supplementary Table 2).

### Comprehensive identification of TUG1-interacting proteins using CARPID

**Fig. 3:**
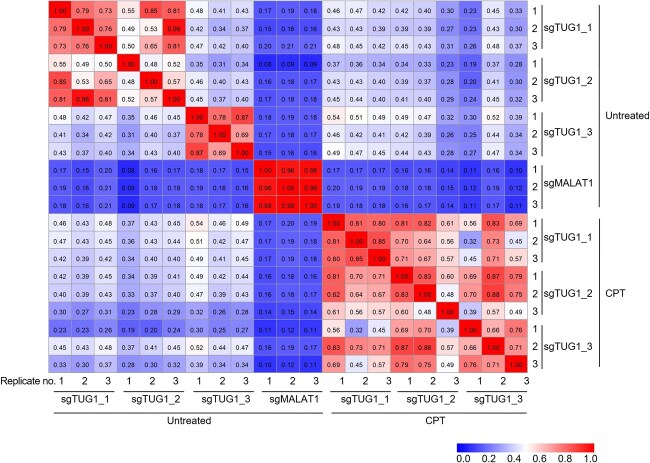
**CARPID reveals reproducible and condition-dependent TUG1 interactomes.** Pearson correlation heatmap of protein abundance (calculated by subtracting the signal values obtained from sgNoTarget-transfected cells from those of TUG1 or MALAT1 sgRNA-transfected cells) detected by MS. Three independent replicate experiments are presented. The colour bar indicates the correlation coefficient (*r*).

Building on our optimized CARPID system, we systematically identified TUG1-interacting proteins using three different sgRNAs (sgTUG1_1, sgTUG1_2, sgTUG1_3) targeting spatially distant single-stranded regions of TUG1. To minimize non-specific signals resulting from stochastic binding, we subtracted the MS signal values obtained from sgNoTarget-transfected cells from those of TUG1 sgRNA-transfected cells. To evaluate the reproducibility of the CARPID assay, we conducted biological triplicates for each of the three sgRNAs targeting TUG1. Correlation analysis (Fig. 3) showed that replicate-level reproducibility was consistent across all sgTUG1 conditions, with the lowest Pearson *r* values being 0.73, 0.48 and 0.69 for sgTUG1_1, _2 and _3, respectively. When comparing across the three TUG1-targeting sgRNAs, sgTUG1_1 and sgTUG1_2 showed relatively high correlation with each other, whereas sgTUG1_3 exhibited lower correlation with both, suggesting that it may enrich for a partially distinct subset of TUG1-associated proteins (Fig. 3).

**Fig. 4:**
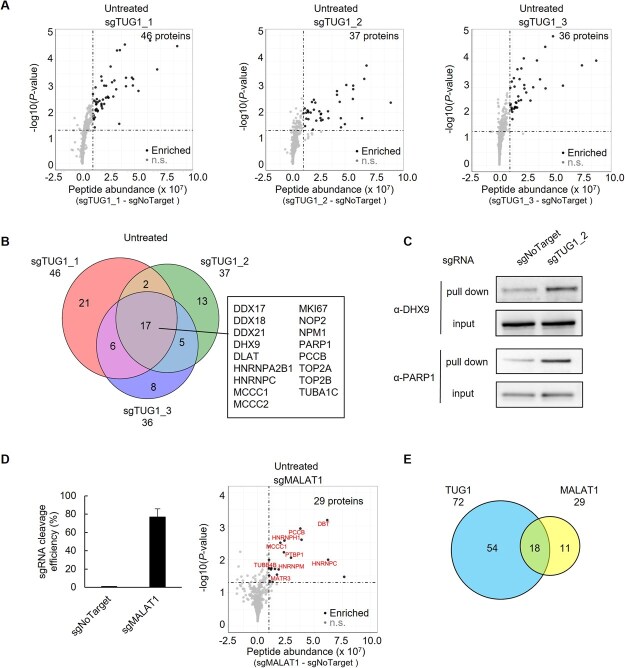
**Identification of TUG1-interacting proteins using CARPID.** (A) Volcano plots showing significantly enriched proteins from CARPID using sgTUG1_1 (left), sgTUG1_2 (middle) and sgTUG1_3 (right). The x-axis shows differences in protein abundance versus sgNoTarget; the y-axis indicates –log_10_ of Benjamini–Hochberg-adjusted *P*-values (two-sided, rank product, *n* = 3). Black dots: significant; n.s.: not significant. (B) Venn diagram showing overlap of enriched proteins from the three TUG1 sgRNAs in untreated cells. Common proteins are listed in the black box. (C) Western blot for DHX9 and PARP1 in input and CARPID pulldown samples from sgNoTarget or sgTUG1_2 transfected cells. (D) Left, RT-qPCR of MALAT1 sgRNA cleavage efficiency versus sgNoTarget. Mean ± SD, *n* = 3. Right, volcano plot of proteins enriched by sgMALAT1. Axes and statistical tests as in (A). Previously reported MALAT1-interactors are labeled in the plot. (E) Venn diagram comparing CARPID-identified proteins for TUG1 and MALAT1. TUG1-associated proteins include all non-redundant hits from the three sgRNAs.

Using the triplicate MS data, we identified 46, 37 and 36 proteins that were consistently and significantly enriched proteins in cells transfected with sgTUG1_1-, sgTUG1_2- and sgTUG1_3-expressing vectors, respectively. These proteins exhibited robust enrichment across all replicates, with high abundance and statistical significance, indicating that they represent high-confidence TUG1-interacting proteins (see Materials and Methods for statistical criteria) (Fig. 4A, Supplementary Table 3). Despite targeting distinct binding sites, all three sgRNAs consistently detected 17 proteins (Fig. 4B). Among these, DHX9 is a DEAD/DEAH-box RNA helicase whose interaction with TUG1 has been previously demonstrated (*20*), and its role in R-loop resolution is well established (*35*–*38*). In addition to DHX9, we identified other DEAD/DEAH-box RNA helicases, including DDX17, DDX18 and DDX21. DDX17 binds and resolves R-loops, and promotes replication fork restart (*39*). DDX18 removes R-loops at DNA damage sites and endogenous R-loop prone regions (*40*). DDX21 resolves R-loops when RNA polymerase II is stalled at transcription sites (*41*). Meanwhile, poly (ADP-ribose) polymerase 1 (PARP1) was also detected. It directly binds to R-loops and activates its ADP-ribosylation activity, which modulates RNA helicases such as DHX9 and DDX18, thereby promoting R-loop resolution (*35**,*  *40**,*  *42*). Western blot analysis confirmed the enrichment of DHX9 and PARP1 in CARPID pull-downs targeting TUG1 (Fig. 4C).

To evaluate the specificity of the CARPID assay, we also applied the sgRNA approach to identify proteins that interact with another well-characterized lncRNA MALAT1 (*25**,*  *43*) (Fig. 4D, Supplementary Table 4). Replicate-level correlation for MALAT1-targeting sgRNAs was high (*r* ≥ 0.96), whereas the correlation coefficients between MALAT1- and TUG1-targeting sgRNAs were very low (*r* ≤ 0.21), supporting the specificity of the captured interactomes (Fig. 3). We identified 29 proteins that interacted with MALAT1 (Supplementary Table 5). Comparative analysis of the TUG1 and MALAT1 interactomes revealed that 18 out of the 72 proteins identified using the three TUG1-targeting sgRNAs overlapped with MALAT1-associated proteins (Fig. 4E, Supplementary Table 6).

### CPT treatment enhances associations with RNA/DNA hybrid-interacting proteins

Next, we conducted CARPID in CPT-treated cells to investigate changes in TUG1-associated proteins under replication stress conditions (Supplementary Table 7). Correlation analysis under CPT-treated conditions (Fig. 3) showed that replicate-level reproducibility remained consistent across all sgTUG1 conditions, with the lowest Pearson *r* values being 0.80, 0.48 and 0.66 for sgTUG1_1, _2 and _3, respectively. Interestingly, in contrast to the untreated condition, correlations among the three sgRNAs increased, suggesting a more conserved pattern of TUG1-protein interactions across different regions of the transcript in response to R-loop accumulation (Fig. 3).

**Fig. 5:**
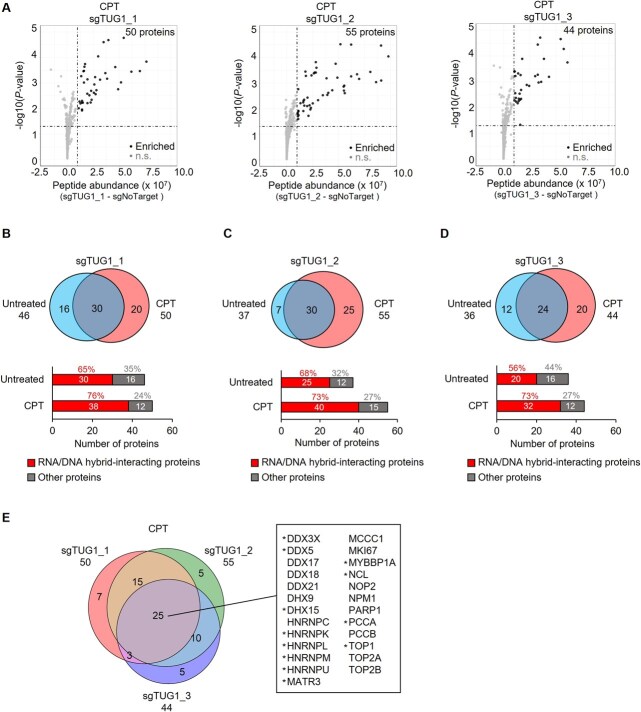
**CPT-induced replication stress increases TUG1-protein interactions identified by CARPID.** (A) Volcano plots showing significantly enriched proteins identified with sgTUG1_1 (left), sgTUG1_2 (middle) and sgTUG1_3 (right) in HEK293T cells treated with 2 μM CPT for 30 min. The x-axis shows protein abundance differences versus sgNoTarget; the y-axis shows -log_10_ of Benjamini–Hochberg-adjusted *P*-values (rank product, two-sided, *n* = 3). Black dots: significant; n.s.: not significant. (B) Comparative analysis for sgTUG1_1. Top, Venn diagram of enriched proteins under untreated versus CPT-treated conditions. Bottom, bar graph showing the number of reported RNA/DNA hybrid-binding proteins among enriched proteins. (C, D) As in (B), for sgTUG1_2 (C) and sgTUG1_3 (D). (E) Venn diagram of proteins enriched by all three sgRNAs in CPT-treated cells. Common proteins are listed in the black box. Twelve newly identified CPT-specific TUG1 interactors are marked with asterisks (*).

CPT exposure increased the number of TUG1-interacting proteins compared to untreated conditions, identifying 50, 55 and 44 consistently and significantly enriched proteins for sgTUG1_1, sgTUG1_2 and sgTUG1_3, respectively (Fig. 5A, Supplementary Table 8). Comparative analysis between untreated and CPT-treated conditions revealed substantial overlap in the identified proteins (Fig. 5B–D). Among the proteins interacting with TUG1 under basal conditions, 65% (30 out of 46) of those identified with sgTUG1_1 were previously reported as RNA/DNA hybrid-interacting proteins based on comprehensive studies (Supplementary Table 3) (*35**,*  *44**,*  *45*). This proportion increased to 76% (38 out of 50) following CPT treatment (Fig. 5B, Supplementary Table 8). A similar trend was observed for sgTUG1_2 and sgTUG1_3, where the fraction of RNA/DNA hybrid-interacting proteins increased from 68 to 73% and from 56 to 73%, respectively, upon CPT treatment (Fig. 5C and D, Supplementary Tables 3 and 8). These results suggest that TUG1 interacts with RNA/DNA hybrid-binding proteins under steady-state conditions and that CPT-induced replication stress, which promotes R-loop accumulation, enhances these interactions.

Among the proteins identified under CPT treatment, 25 were consistently detected across all three sgRNAs. This set included 13 of the 17 proteins previously identified under untreated conditions, while 12 were newly identified following CPT treatment (Fig. 5E). Following CPT treatment, the number of DEAD/DEAH-box RNA helicases interacting with TUG1 increased from four to seven. Among three additional RNA helicases, DDX3X resolves R-loops by directly degrading RNA/DNA hybrids and enhancing RNaseH2 activity (*46*). DDX5 promotes R-loop degradation by assisting RNA nuclease XRN2 in removing RNA strands (*47*). Although DHX15 has not been directly implicated in R-loop resolution, comprehensive studies have identified it as an RNA/DNA hybrid-interacting protein (*35**,*  *44**,*  *45*).

In addition, the number of heterogeneous nuclear ribonucleoproteins (HNRNPs) identified increased from two to five. HNRNPs are a family of RBPs that regulate various aspects of RNA metabolism, including splicing, stability, transport and translation (*48*). Among them HNRNPU has been reported to suppress excessive R-loop accumulation (*49*). Furthermore, in addition to TOP2A and TOP2B, CPT treatment also increased the association of TUG1 with TOP1. Topoisomerases are critical regulators of DNA topology and play crucial roles in R-loop homeostasis (*50*–*52*).

### CPT-induced changes in the TUG1 interactome reveal recruitment of RNA processing and genome stability factors

**Fig. 6:**
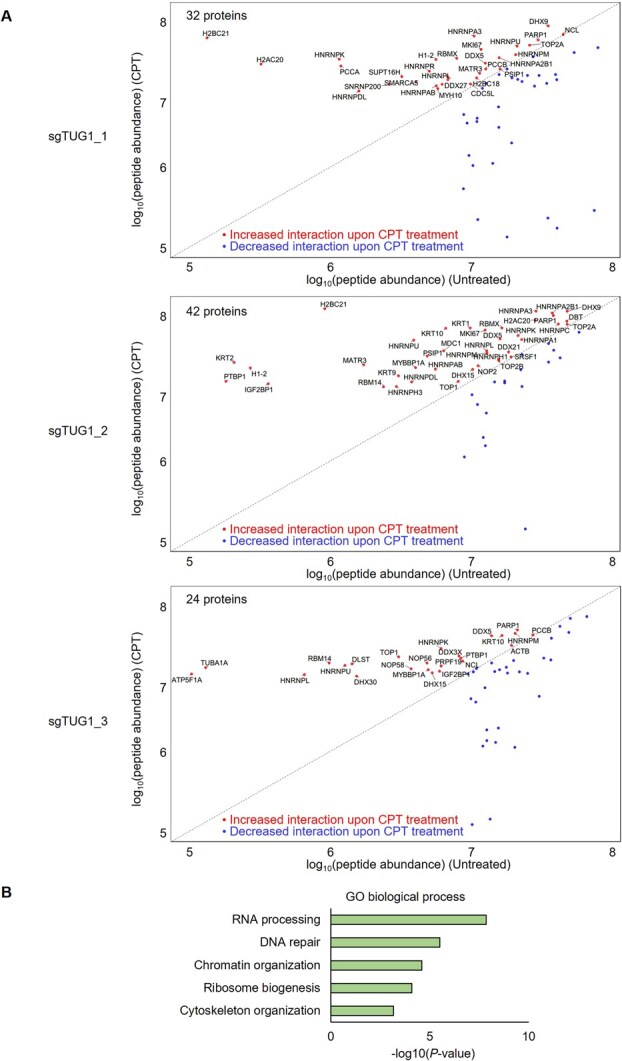
**CPT-induced expansion of the TUG1 interactome and functional enrichment analysis.** (A) Scatter plots comparing peptide abundance of TUG1-interacting proteins in untreated (x-axis) and CPT-treated (y-axis) cells using sgTUG1_1 (top), sgTUG1_2 (middle) and sgTUG1_3 (bottom). Dots representing proteins with increased or decreased interaction are shown; proteins with increased interaction are labelled in the plot. (B) GO analysis of 61 proteins with increased binding to TUG1 after CPT treatment. The x-axis shows -log_10_(*P*-value) of enriched terms; the y-axis lists the top five GO biological processes common to all three sgRNAs.

We next aimed to identify proteins that exhibited increased interaction with TUG1 following CPT treatment. For each sgRNA, we extracted proteins whose association with TUG1 was enhanced under CPT-induced R-loop formation. This approach identified factors that are actively recruited in response to replication stress. In total, 32, 42 and 24 proteins exhibited increased interaction across the three sgRNAs (Fig. 6A), and after removing duplicates, 61 unique proteins remained. To gain further insight into their functional roles, we performed GO analysis (Fig. 6B, Table I). GO analysis revealed that RNA processing was the most enriched biological process among CPT-induced TUG1 interactors (Fig. 6B, Table I). This category includes proteins involved in RNA maturation, modification, splicing, transport and degradation, such as DEAD/DEAH-box helicases and HNRNPs. In the category of chromatin organization, the most enriched proteins under CPT treatment were Histone H2B variants, followed by Histone H2A variants (Table I). These are not canonical histones but variants that showed a marked increase in abundance after CPT exposure (e.g. from 0.04 × 10^7^ to 4.91 × 10^7^ for H2B variants, and from 0.83 × 10^7^ to 3.09 × 10^7^ for H2A variants) (Table I). Among the H2A variants identified, H2AX is most likely included, suggesting that the accumulation of γH2AX at sites of CPT-induced R-loops or R-loop–associated DNA damage (*53**,*  *54*) may account for its detection. However, the specific variant could not be conclusively determined due to shared peptide sequences among H2A variants. In contrast, none of the identified H2B variants have been previously reported to be associated with R-loops or DNA damage, and their functional significance remains unclear. Additionally, mediator of DNA damage checkpoint 1 (MDC1) facilitates chromatin remodelling at DNA damage sites, thereby promoting access of repair factors to damaged chromatin (*55*), while SWI/SNF-related matrix-associated actin-dependent regulator of chromatin subfamily A member 5 (SMARCA5) functions as an ATP-dependent chromatin remodeller that modulates nucleosome positioning and facilitates chromatin dynamics (*56*). Notably, MDC1 and SMARCA5 are classified under both the chromatin organization and DNA repair GO categories (Table I). Collectively, these representative proteins illustrate that TUG1 dynamically reorganizes its protein interactions in response to replication stress, potentially engaging factors involved in RNA metabolism and genome stability maintenance.

**Table I:** GO analysis of proteins exhibiting increased interaction to TUG1 following CPT treatment

**No.**	**Gene symbol**	**Protein name**	**Average peptide abundance across three sgRNAs (×10** ^**7**^**)**	
			**Untreated condition**	**CPT-treated condition**
**RNA processing**				
1	DHX9	DEAH-box helicase 9	5.33	7.18
2	HNRNPA3	Heterogeneous nuclear ribonucleoprotein A3	1.75	4.94
3	HNRNPA2B1	Heterogeneous nuclear ribonucleoprotein A2/B1	3.29	3.96
4	DDX5	DEAD-box helicase 5	1.64	3.19
5	HNRNPK	Heterogeneous nuclear ribonucleoprotein K	0.24	3.08
6	HNRNPM	Heterogeneous nuclear ribonucleoprotein M	2.09	3.04
7	HNRNPU	Heterogeneous nuclear ribonucleoprotein U	1.02	3.01
8	RBMX	RNA-binding motif protein, X chromosome	1.04	2.79
9	HNRNPA1	Heterogeneous nuclear ribonucleoprotein A1	1.64	1.85
10	HNRNPL	Heterogeneous nuclear ribonucleoprotein L	0.77	1.79
11	PSIP1	PC4 and SFRS1-interacting protein	0.91	1.46
12	SRSF1	Serine/arginine-rich splicing factor 1	0.78	1.35
13	DHX15	DEAH-box helicase 15	1.04	1.26
14	NOP58	Nucleolar protein 58	0.82	1.19
15	PTBP1	Polypyrimidine tract-binding protein 1	0.90	1.05
16	RBM14	RNA binding motif protein 14	0.69	1.04
17	HNRNPR	Heterogeneous nuclear ribonucleoprotein R	0.26	0.97
18	CDC5L	Cell division cycle 5-like protein	0.68	0.72
19	PRPF19	Pre-mRNA-processing factor 19	0.62	0.71
20	SNRNP200	U5 small nuclear ribonucleoprotein 200 kDa helicase	0.24	0.69
21	DDX27	DEAD-box helicase 27	0.34	0.61
22	HNRNPH3	Heterogeneous nuclear ribonucleoprotein H3	0.35	0.39
**Chromatin organization**				
1^a^	H2BC13	Histone H2B type 1-L	0.04	4.91
	H2BC18	Histone H2B type 2-F		
	H2BC21	Histone H2B type 2-E		
	H2BK1	Histone H2B type 2-KI		
2^a^	H2AC18	Histone H2A type 3	0.83	3.09
	H2AC20	Histone H2A type 2-C		
	H2AC21	Histone H2A type 2-B		
	H2AX	Histone H2AX		
	H2AZ2	Histone H2A.V		
3	HNRNPU	Heterogeneous nuclear ribonucleoprotein U	1.02	3.01
4	MDC1	Mediator of DNA damage checkpoint protein 1	1.24	1.74
5	H1–2	Histone H1.2	0.28	1.47
6	PSIP1	PC4 and SFRS1-interacting protein	0.91	1.46
7	TOP1	DNA topoisomerase 1	1.22	1.34
8	RBM14	RNA binding motif protein 14	0.69	1.04
9	SUPT16H	SPT16 homologue, facilitates chromatin remodelling subunit	0.25	0.88
10	SMARCA5	SWI/SNF-related matrix-associated actin-dependent regulator of chromatin subfamily A member 5	0.63	0.84
^a^Histone variants with shared peptide sequences are listed together due to their indistinguishable MS profiles.				
**DNA repair**				
1	PARP1	Poly (ADP-ribose) polymerase 1	4.32	5.20
2	TOP2A	DNA topoisomerase 2-alpha	4.21	4.40
3	MDC1	Mediator of DNA damage checkpoint protein 1	1.24	1.74
4	SMARCA5	SWI/SNF-related matrix-associated actin-dependent regulator of chromatin subfamily A member 5	0.63	0.84
5	PRPF19	Pre-mRNA-processing factor 19	0.62	0.71

## Discussion

In this study, we significantly expand our previous findings by identifying a comprehensive interactome of TUG1 under physiological conditions. Our previous study was the first to demonstrate that under replication stress, TUG1 is rapidly upregulated via ATR-CHK1 signalling and interacts with DHX9, playing a crucial role in resolving R-loops formed at replication forks in cancers (*20*). In line with that, the current CARPID-based analysis in HEK293T cells also identified DHX9 as a major interactor, confirming the robustness of this interaction. In addition, CARPID identified a broader set of proteins, ~70% of which are known to interact with RNA/DNA hybrids, including several RNA helicases and DNA repair factors. These differences likely reflect both the higher sensitivity of CARPID in capturing transient and proximity-based interactions, and the use of different cell types, which may influence the composition of the TUG1 interactome.

CARPID provided a robust and unbiased approach for profiling lncRNA interactomes, as it enables the capture of transient and condition-specific interactions in living cells. This is particularly advantageous for studying dynamic interactomes under stress conditions, where RNA-protein associations are rapidly remodelled. Recent studies have similarly applied CARPID to map interactions with circular RNA, another class of non-coding RNAs (*57*–*59*). Given that lncRNAs exert their functions through context-dependent interactions with RBPs, CARPID and Cas13-based proximity labelling methods are well suited for studying these interactions.

Figure 3 presents the region-specific patterns of protein association along the TUG1 transcript. Since RNA molecules adopt secondary and tertiary structures that can influence protein binding (*60**,*  *61*), the observed differences among sgRNAs may be influenced by the 3D folding of TUG1, which could bring certain regions into proximity while separating others. Notably, upon CPT treatment, the correlation coefficients among all three TUG1-targeting sgRNAs increased, indicating a more conserved TUG1 interactome under replication stress. This may reflect structural changes in TUG1 or stress-induced remodelling of its associated protein complexes, resulting in increased accessibility or uniformity across the transcript. Taken together, these results demonstrate that protein interactions with the lncRNA TUG1 become more coordinated across its transcript under replication stress—a novel and previously unrecognized feature of lncRNA-mediated R-loop regulation.

To highlight the most consistent interactions, we defined a core set of 17 proteins consistently identified across all three sgRNAs under basal conditions, which expanded to 25 proteins following CPT treatment. These proteins may form stable and high-affinity interactions with TUG1 or reside in close spatial proximity, allowing efficient biotinylation from all sgRNA-binding sites within the 10-nm labelling radius of BASU biotin ligase (*25*). The abundant detection of DHX9 (5.33 × 10^7^ and 7.18 × 10^7^ in peptide abundance under untreated and CPT-treated conditions, respectively; Table I) supports its preferential interaction with TUG1, consistent with prior evidence. One limitation of the CARPID method is the inherent difficulty in biotinylating certain proteins due to steric hindrance, competition among proximal proteins (*25*). For instance, the lack of RPA, despite our previous demonstration of its direct interaction with TUG1, might be due to these technical constraints.

Nonetheless, this approach effectively identified multiple DEAD/DEAH-box RNA helicases as the most prominent TUG1 interactors, particularly under CPT-induced replication stress. Under basal conditions, DHX9, DHX17, DHX18 and DHX21 were consistently identified across all three sgRNAs (Fig. 4B). Following CPT treatment, three additional RNA helicases—DDX3X, DDX5 and DHX15—were identified as TUG1 interactors (Fig. 5E). The observed increase in TUG1-interacting proteins following CPT treatment may be explained by two reasons. First, CPT-induced upregulation of TUG1 (Fig. 1B) likely increases the probability of capturing more interacting proteins. Second, CPT treatment may induce conformational changes in TUG1 or alter its protein partners, potentially enabling new interactions to form. Together, these changes may account for the expanded TUG1 interactome observed under replication stress conditions.

Still, it remains unclear how each helicase is selectively utilized for R-loop resolution in the genome. Given previous reports indicating that lncRNAs often function as molecular scaffolds (*62**,*  *63*), it is plausible that TUG1 similarly recruits helicases to specific genomic loci prone to R-loop formation. This could enhance the efficiency of R-loop removal by concentrating helicases at sites of R-loop formation. Additionally, RNA has been shown to regulate helicase activity through allosteric modulation (*64*) or by promoting protein–protein interactions (*60**,*  *65*). TUG1 may be involved in similar mechanisms. While the specific contribution of each factor to distinct R-loop subsets and their potential cooperation with TUG1 remain to be elucidated, our findings provide a foundation for future functional studies aimed at dissecting these mechanisms. Future studies employing genome-wide approaches (such as ChIP-seq or DRIP-seq) combined with CARPID will be valuable to clarify whether TUG1 interacts with specific genomic regions and acts collaboratively with RBPs to resolve R-loops. Future studies combining genome-wide approaches (such as ChIP-seq or DRIP-seq) with CARPID will be valuable for determining whether TUG1 interacts with specific genomic regions and cooperates with RBPs to resolve R-loops. Given our prior demonstration of the essential roles of TUG1 in cancer cells (*20*), applying CARPID to cancer cell models will enable characterization of the dynamic TUG1 interactome under cancer-specific replication stress.

Overall, these findings establish TUG1 as a key regulatory component of the cellular response to R-loop accumulation. By dynamically engaging RNA helicases, PARP1, HNRNPs and topoisomerases, TUG1 likely facilitates multiple aspects of R-loop resolution, including their recognition, unwinding and removal, as well as subsequent DNA repair and chromatin remodelling.

## Supplementary Material

Web_Material_mvaf042
